# Enhancing Chiroptical Selectivity and Device Stability of Chiral Perovskite Photodetectors Via Interfacial AlF_3_ Passivation

**DOI:** 10.1002/smtd.71019

**Published:** 2026-09-07

**Authors:** Hyejin Lim, Su Hye Jeong, Chae Bin Lee, Yousang Won, Seungwon Lee, Inho Song, Wooseok Song, Ki‐Seok An, Sungwoong Park, Seungju Lee, Soonmin Yim, Yoon Ho Lee, Saewon Kang

**Affiliations:** ^1^ Thin Film Materials Research Center Korea Research Institute of Chemical Technology (KRICT) Daejeon Republic of Korea; ^2^ Department of Materials Science and Engineering Sungshin Women's University Seoul Republic of Korea; ^3^ Samsung Electronics Co., Ltd Suwon Republic of Korea; ^4^ Department of Chemical Engineering Chung‐Ang University (CAU) Seoul Republic of Korea

**Keywords:** atomic layer deposition, chiral perovskite layer, perovskite photodetector, stability

## Abstract

Chiral halide perovskites are promising materials for circularly polarized light (CPL) optoelectronics owing to their strong light absorption, long carrier diffusion lengths, and intrinsic chiroptical activity. However, most reported CPL perovskite devices still suffer from limited chiroptical selectivity and poor operational stability, hindering practical applications. Here, we report a facile interfacial passivation strategy using an atomic layer deposition (ALD)‐grown AlF_3_ film for high‐performance and stable CPL photodetectors (PDs). The dense AlF_3_ underlayer modifies the local interfacial environment of the perovskite, promotes grain growth, and reduces defect sites in the chiral perovskite films. Consequently, the AlF_3_‐based films exhibit enhanced apparent chiroptical properties, with circular dichroism (CD) and circularly polarized photoluminescence (CPL‐PL) *g*‐factors increasing by approximately 3.8‐fold and 2‐fold, respectively, compared with pristine films. CPL PDs fabricated on the AlF_3_ underlayer achieve a photoresponsivity of 54 mA W^−1^, a specific detectivity of 1.68 × 10^11^ Jones, and a photocurrent *g*‐factor of 0.48, representing an 84% enhancement over pristine devices. Interfacial AlF_3_ passivation also significantly improves device durability, providing a *T*_70_ operational lifetime more than 9.3 times longer under ambient conditions. This work demonstrates an effective interface‐engineering strategy for simultaneously enhancing chiroptical selectivity, device performance, and operational stability in CPL perovskite optoelectronics.

## Graphical abstract

An ALD‐grown AlF_3_ underlayer promotes grain growth, defect passivation, and improved crystalline ordering in chiral perovskite films. The resulting films exhibit enhanced chiroptical properties, superior circularly polarized light photodetector performance, and improved stability, establishing an inorganic interface‐engineering strategy broadly applicable to perovskite chiral optoelectronic devices.

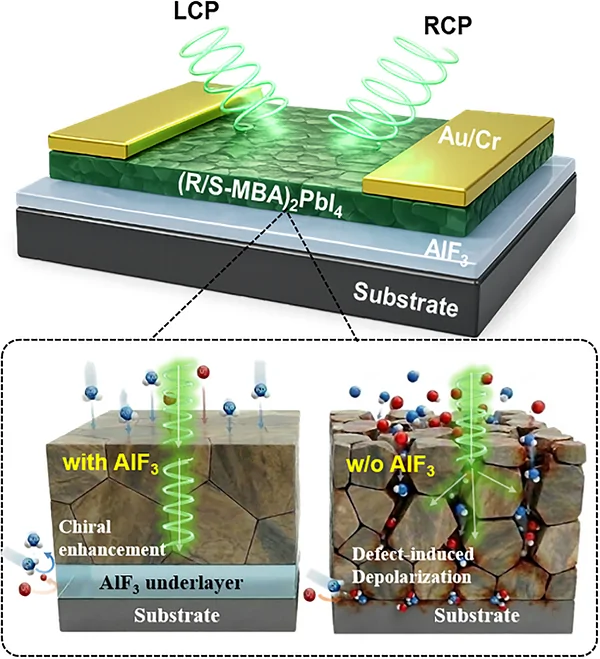

## 1 Introduction

Circularly polarized light (CPL) detection and emission are emerging as key capabilities for next‐generation photonic technologies, including quantum information processing, spin‐encoded optical communication, information storage, 3D displays, and polarization‐resolved bio‐imaging [1, 2, 3, 4]. Circular polarization‐sensitive photodetectors (PDs) and emitters that can convert the intensity and handedness of CPL as distinguishable electrical or optical outputs are essential for these applications [5, 6, 7, 8].

Among various chiral material systems, chiral halide perovskites have attracted considerable attention as CPL optoelectronic semiconductors, owing to their tunable optical bandgaps, low trap densities, strong light absorption coefficients, and long carrier diffusion lengths [9, 10, 11, 12, 13, 14]. The incorporation of chiral organic ligands into the perovskite lattice enables efficient chirality transfer from the organic to the inorganic framework, giving rise to circular dichroism (CD) and circularly polarized photoluminescence (CPL‐PL) responses that are directly applicable for CPL detection and emission [15, 16]. These combined properties make chiral halide perovskites one of the most promising material platforms for next‐generation CPL optoelectronics. However, achieving high‐performance and stable CPL devices based on chiral perovskites remains a significant challenge. Most reported chiral perovskite CPL optoelectronics show a trade‐off between chiroptical selectivity and key device figures of merit such as photoresponsivity and detectivity, and poor operational stability under ambient conditions further limits their practical use [17, 18, 19, 20].

Solution‐processed chiral perovskite films typically suffer from high densities of grain boundary defects, surface ions, and morphological inhomogeneities such as pinholes, all of which suppress chiroptical response by degrading excitonic coherence and reduce photodetector performance by promoting non‐radiative recombination and polarization‐insensitive photocurrent [21, 22, 23]. These structural imperfections also act as preferential sites for moisture‐driven decomposition, directly linking poor film morphology to poor operational stability [24, 25]. Conversely, films with enlarged grains and reduced grain boundary defects can better preserve the chirality transferred from the chiral organic ligands to the inorganic framework, directly enhancing the dissymmetry (*g*) factors of both absorption and emission [26, 27, 28]. Improving film quality in a way that simultaneously enhances chiroptical selectivity, device performance, and stability is therefore the central materials challenge for chiral perovskite CPL optoelectronics.

To improve perovskite film quality, various interface‐engineering strategies have been developed [29], including self‐assembled monolayers (SAMs) [30], ultrathin organic interlayers [31], high‐polarity molecular or polymer coatings [32, 33], and 2D/3D heterostructure capping layers [34], and have shown success in regulating nucleation [35], improving crystallinity, passivating defects, and suppressing non‐radiative recombination. However, these approaches rely predominantly on organic or hybrid materials that are susceptible to thermal and chemical degradation under device operating conditions [36, 37]. In contrast, inorganic interlayers exhibit superior resistance to thermal and chemical degradation and can establish dense, chemically stable interfaces with perovskite films, making them promising candidates for simultaneously mitigating interfacial defects and improving environmental stability. Therefore, the development of an inorganic interface‐engineering approach that provides conformal and chemically robust coverage together with effective defect passivation is highly desirable to simultaneously overcome the chiroptical, optoelectronic, and stability limitations of chiral perovskite CPL devices. Moreover, there is no study demonstrating a single‐step strategy capable of simultaneously enhancing chiroptical selectivity, device performance, and operational stability in chiral perovskite CPL optoelectronics.

Here, we report an underlayer passivation strategy based on atomic layer deposition (ALD)‐grown fluorinated inorganic AlF_3_ films and demonstrate CPL perovskite PDs with high performance and enhanced stability. Chiral perovskite films fabricated on dense ALD‐grown AlF_3_ layers show a modified local interfacial environment, together with larger grain sizes and reduced defect sites, which are accompanied by enhanced chiroptical properties. The introduction of the AlF_3_ interlayer led to approximately 3.8‐fold and 2‐fold increases in the CD and CPL‐PL *g*‐factors, respectively, compared with those of the pristine films. Moreover, AlF_3_‐based CPL perovskite PDs exhibit a photoresponsivity (*R*) of 54 mA W^−1^, a specific detectivity (*D* ^*^) of 1.68 × 10^11^ Jones, and a photocurrent *g*‐factor of 0.48, corresponding to an 84% increase in the photocurrent *g*‐factor compared with pristine devices. Furthermore, the improved crystallinity of the perovskite film induced by the AlF_3_ layer, together with its passivation effect, markedly enhances the stability of the chiral perovskite layer, yielding a *T*_70_ value for working stability in ambient conditions that is more than 9.3 times higher than that of the pristine PDs. This work not only paves the way for high‐performance and stability chiral perovskite PDs, but also offers a promising pathway toward interface‐engineered perovskite platforms for a wide variety of electronic and optoelectronic devices.

## 2 Results and Discussion

### 2.1 Chiral Perovskite Films With ALD AlF_3_ Underlayer

Figure 1a presents the overall device architecture of the chiral perovskite CPL PDs with the AlF_3_ underlayer and fabrication scheme of AlF_3_ film. The device consists of a Si substrate/AlF_3_/(*R/S*‐MBA)_2_PbI_4_/Cr/Au stack (MBA: methylbenzylammonium iodide), where AlF_3_ thin films were deposited by thermal ALD using trimethylaluminum (TMA) and HF–pyridine as precursors at 125°C. The ALD process enables angstrom‐level thickness control and conformal, pinhole‐free coverage, which are critical for providing a uniform nucleation template for the perovskite film [38, 39]. The top‐view and cross‐sectional scanning electron microscope (SEM) images of the AlF_3_ film confirm a dense, uniform surface morphology with no visible pinholes (Figure S1). Figure 1b schematically illustrates the proposed mechanism of the chirality and stability enhancement induced by the AlF_3_ underlayer. The ALD‐grown AlF_3_ surface provides a chemically homogeneous, fluorine‐rich interface, which may modify the local interfacial environment and influence perovskite crystallization. This interfacial effect may reduce excessive nucleation and promote lateral grain growth [40, 41]. The resulting enlargement of grain size reduces the total grain boundary area, a major source of trap states and non‐radiative recombination centers in solution‐processed perovskite films [42]. In pristine films, abundant grain boundaries, pinholes, and surface defects can act as scattering and depolarization centers that disrupt the ordered chiral structure of the inorganic framework, thereby weakening the chiroptical response of the film. In contrast, the large and well‐ordered grains grown on the AlF_3_ surface are expected to better preserve the chirality transferred from the chiral organic ligands to the inorganic framework and enhancing film stability. Moreover, the AlF_3_ film acts as a protective layer that prevents moisture from penetrating into the perovskite film from the underlying substrate [43]. Based on this proposed mechanism, the *g*‐factor and stability of the AlF_3_‐based films are expected to increase along with the improved film morphology [27].

**FIGURE 1:**
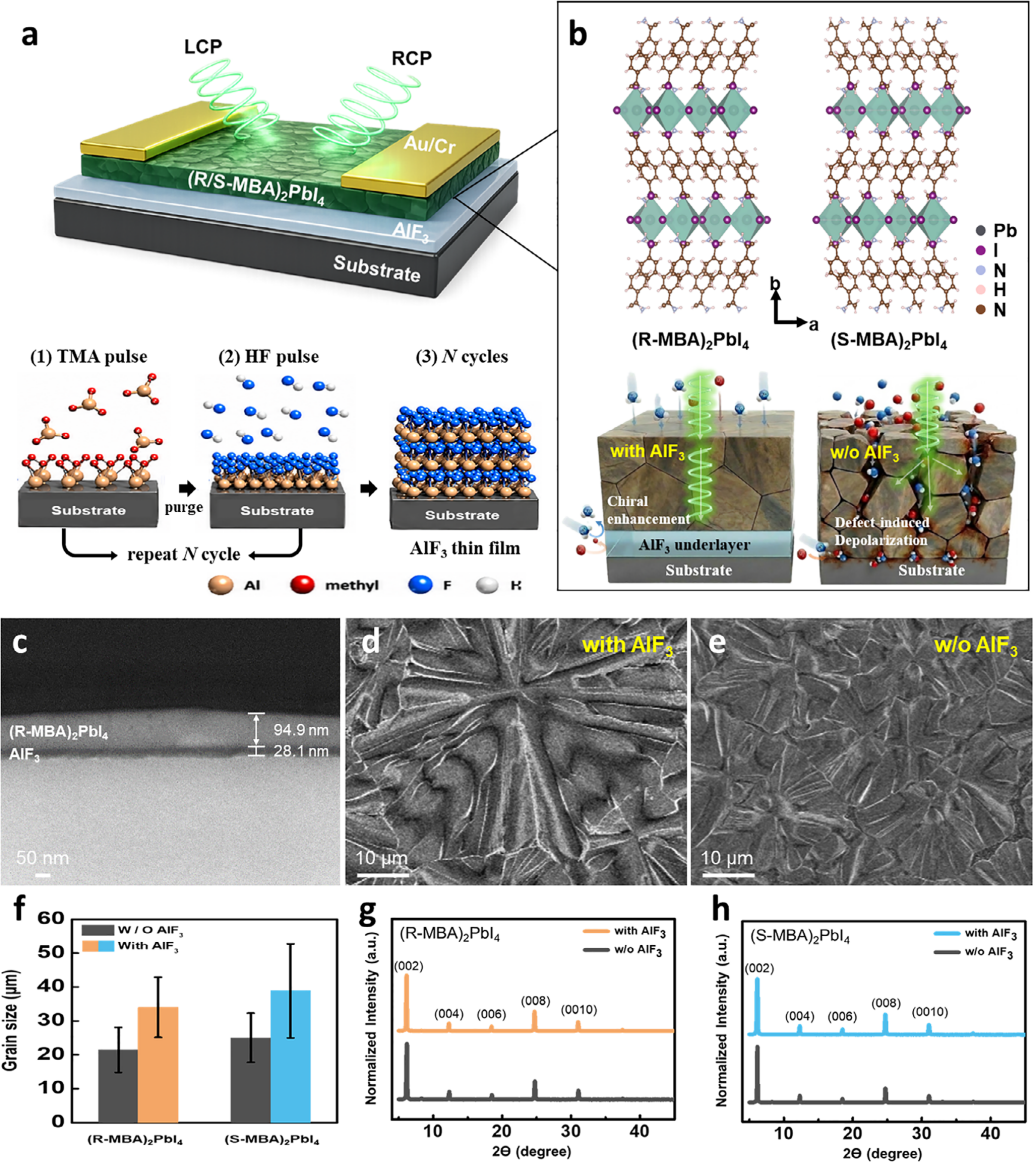
Chiral perovskite CPL PDs with AlF_3_ underlayer. (a) Schematic of the CPL PDs device stack (Si/SiO_2_/AlF_3_/(*R/S*‐MBA)_2_PbI_4_/Cr/Au). Inset: schematic of AlF_3_ deposition by thermal ALD using TMA and HF–pyridine precursors. (b) Schematic images of (*R*‐MBA)_2_PbI_4_ and (*S*‐MBA)_2_PbI_4_ crystal structures (top) and schematic illustration of the proposed mechanism of grain size and defect passivation enhancement in chiral perovskite films with and without the AlF_3_ underlayer (bottom). (c) Cross‐sectional SEM image of (*R*‐MBA)_2_PbI_4_ films CPL PDs with AlF_3_ underlayer. (d, e) Top‐view SEM images of (*R*‐MBA)_2_PbI_4_ films deposited (d) with and (e) without 30 nm AlF_3_. (f) Average grain sizes of (*R*‐MBA)_2_PbI_4_ and (*S*‐MBA)_2_PbI_4_ films with and without AlF_3_. (g, h) XRD patterns of (*R*‐MBA)_2_PbI_4_ (g) and (*S*‐MBA)_2_PbI_4_ (h) films with and without 30 nm AlF_3._

The cross‐sectional SEM image of the Si substrate/AlF_3_ (30 nm)/(*R*‐MBA)_2_PbI_4_ structure clearly shows each layer with sharp interlayer boundaries (Figure 1c), confirming that the AlF_3_ interlayer maintains its integrity throughout the device fabrication process. Chiral (*R*/*S*‐MBA)_2_PbI_4_ films were subsequently deposited on the AlF_3_/Si substrate by spin‐coating a precursor solution in DMF:DMSO (10:1 v/v), followed by thermal annealing at 100°C under nitrogen atmosphere. The top‐view SEM images show that the film on AlF_3_ exhibits denser grain packing with an average grain size of 34 µm, representing a 58% increase over the pristine film on bare Si substrate (Figures 1d,e and Figure S2). The reduction in pinholes is particularly significant for CPL photodetector applications because pinholes not only act as recombination sites that reduce photocurrent, but also introduce polarization‐insensitive conduction pathways that degrade the chiroptical anisotropy factor. The grain size distributions of both (*R*‐MBA)_2_PbI_4_ and (*S*‐MBA)_2_PbI_4_ films are shown in Figure 1f, confirming consistent grain size enhancement for both enantiomers upon AlF_3_ introduction. The average grain size increases with AlF_3_ thickness up to an optimal point and decreases at higher thicknesses, suggesting an optimal underlayer thickness for promoting lateral grain growth (Figure S3). A thickness of 30 nm was therefore selected as the representative condition for device fabrication.

X‐ray diffraction (XRD) patterns of both (*R*‐MBA)_2_PbI_4_ and (*S*‐MBA)_2_PbI_4_ films with and without the AlF_3_ underlayer show predominantly the (002), (004), (006), (008), and (0010) reflections of the 2D layered perovskite structure (Figure 1g,h). The absence of secondary phases or PbI_2_ impurity peaks confirms that the AlF_3_ underlayer does not induce bulk compositional or structural changes in the perovskite film. Films on AlF_3_ show slightly enhanced diffraction peak intensities, consistent with the improved crystallinity observed in SEM.

To further examine the effect of the AlF_3_ underlayer on the chemical environment of the perovskite, additional XPS measurements were performed under conditions optimized to probe the AlF_3_/perovskite interface (Figure S4). Besides the common Pb 4f, I 3d, C 1s, and O 1s signals, Al 2p and F 1s signals were additionally detected in the AlF_3_‐containing sample, with the F 1s peak (∼686.75 eV) mainly attributed to Al–F bonding (Figure S4a,b). The Pb 4f spectra showed no appreciable shift, whereas both I 3d peaks shifted by approximately 0.22 eV toward lower binding energy (Figure S4c,d), suggesting that the AlF_3_ underlayer may influence the local environment around iodide near the interface.

### 2.2 Optical and Chiroptical Properties of Chiral Perovskite Films With AlF_3_ Underlayer

The ultraviolet (UV)‐visible (Vis) absorption spectra and photoluminescence (PL) spectra of the (*R*/*S*‐MBA)_2_PbI_4_ films fabricated on glass substrates without and with the AlF_3_ layers are shown in Figures 2a,b and Figure S5. All perovskite films show absorption and PL peak positions at 501 nm and 512 nm, respectively, consistent with previously reported values [21, 44]. Interestingly, under the same excitation and detection conditions, the perovskite films with AlF_3_ layers has a stronger light absorption and PL intensity than perovskite films without AlF_3_, demonstrating the high crystallinity of the perovskite films with AlF_3_ layers [45, 46]. The enhanced light absorption observed in the perovskite films on AlF_3_‐modified substrates could be initially attributed to additional absorption from the AlF_3_ interlayer. However, AlF_3_ deposited on transparent substrates such as glass generally acts as an antireflection layer (Figure S6), thereby reducing surface reflection and increasing optical transmittance rather than contributing to parasitic absorption. Therefore, the observed increase in absorption is primarily attributed to modifications in the perovskite layer itself.

**FIGURE 2:**
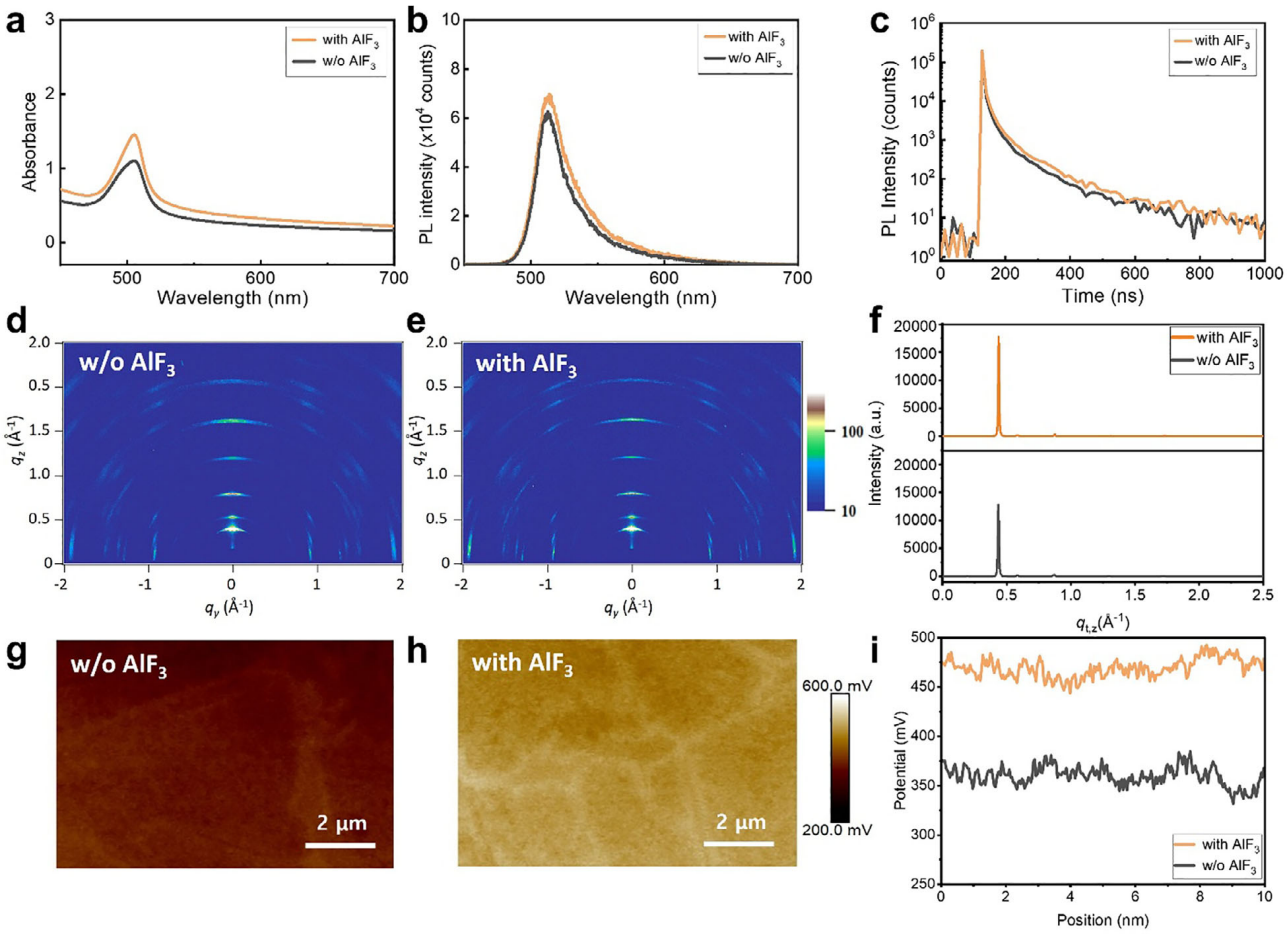
(a,b) (a) UV–vis absorption spectra and (b) PL spectra of (*R*‐MBA)_2_PbI_4_ films without and with an AlF_3_ interlayer. (c) TR‐PL decay curves of (*R*‐MBA)_2_PbI_4_ films without and with an AlF_3_ interlayer. (d,e) 2D GIWAXS patterns of (*R*‐MBA)_2_PbI_4_ films (d) without and (e) with an AlF_3_ interlayer. (f) Corresponding out‐of‐plane GIWAXS line‐cut profiles extracted from the 2D GIWAXS patterns. (g,h) KPFM surface potential maps of (*R*‐MBA)_2_PbI_4_ films (g) without and (h) with an AlF_3_ interlayer. (i) Surface potential line profiles extracted from the corresponding KPFM measurements.

This is further supported by time‐resolved photoluminescence (TR‐PL) for (*R*‐MBA)_2_PbI_4_ and (*S*‐MBA)_2_PbI_4_ as shown in Figure 2c and Figure S7. The perovskite films with AlF_3_ layers show longer lifetime, confirming relatively low non‐radiative recombination of the perovskite. Additional thickness‐dependent TR‐PL measurements of the (*R*‐MBA)_2_PbI_4_ films showed that the PL decay lifetime increased with increasing AlF_3_ thickness up to 30 nm and decreased at 50 nm (Figure S8). The *τ*_1_ values were 8.54, 11.55, 13.13, and 10.69 ns for the 0, 10, 30, and 50 nm AlF_3_ interlayers, respectively. The corresponding *τ*_2_ values were 36.13, 58.58, 72.82, and 51.22 ns, respectively. Both lifetime components reached their maximum values for the 30 nm AlF_3_ interlayer. A similar thickness‐dependent tendency was also observed in the grain‐size distribution shown in Figure S3.

A grazing incidence wide‐angle x‐ray diffraction (GIWAXS) analysis was also employed to investigate the influence of the AlF_3_ film for the crystalline structure of perovskite films. Figure 2d,e show GIWAXS patterns of (*R*‐MBA)_2_PbI_4_ film without and with AlF_3_ films, revealing well‐matched 2D perovskite crystal structures. As shown in Figure 2f, the (*R*‐MBA)_2_PbI_4_ film deposited on the AlF_3_ layer exhibits a substantially higher out‐of‐plane (001) diffraction intensity than the film without AlF_3_, suggesting an increased degree of crystal ordering and a larger population of highly ordered crystalline domains [47, 48]. Such improved structural ordering is expected to suppress structural disorder and defect formation, thereby contributing to the enhanced optical properties [49, 50]. GIWAXS measurements performed at incident angles ranging from 0.2° to 1.1° (Figure S9) revealed consistently stronger diffraction intensities for the films deposited on the AlF_3_ layer than for the control films, suggesting that the enhanced crystal ordering persists throughout the film thickness.

We also used Kelvin probe force microscopy (KPFM) to investigate the energy level properties of perovskite films. Figure 2g,h show the surface potential images of (*R*‐MBA)_2_PbI_4_ films without and with AlF_3_ films. The corresponding KPFM topography images are shown in Figure S10. The perovskite film deposited on the AlF_3_ layer exhibited a contact potential differences (CPD) approximately 100 mV higher than that of the control film without AlF_3_, corresponding to a lower work function, probably due to the reduced density of defect states in the perovskite film (Figure 2i) [51, 52, 53]. The KPFM results for the (*S*‐MBA)_2_PbI_4_ films are also shown in Figure S11, exhibiting a similar trend with an increase in CPD upon the introduction of the AlF_3_ layer.

### 2.3 Chiroptical Properties of Chiral Perovskite Films With AlF_3_ Layer

The chiroptical absorption properties of the chiral perovskite films were examined by CD spectroscopy (Figure 3). According to the Mueller matrix analysis of the CD spectra of measurement system, the main components of the detected CD (also noted as apparent CD) can be written as [54]:

(1) $$CD\;\approx CD_{genuine}+\frac{1}{2}\left(LD^{\prime }\times LB-LB^{\prime }\times LD\right)-LD\times \sin\alpha \times \cos2\theta$$

**FIGURE 3:**
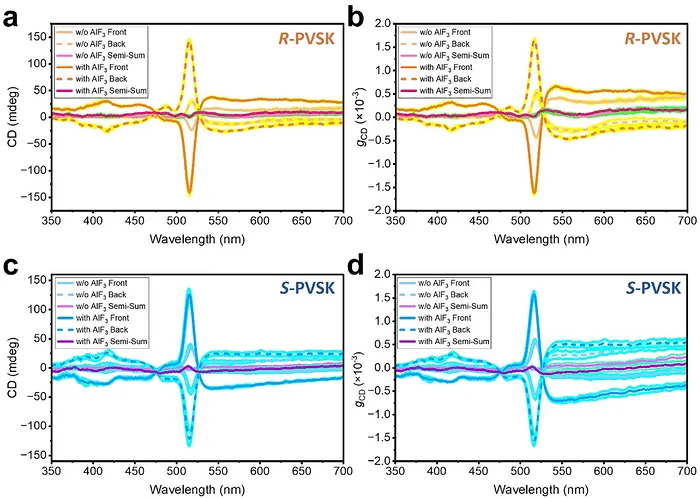
(a,d) (a) CD Spectra, (b) *g*_CD_ spectra of (*R*‐MBA)_2_PbI_4_ and (c) CD Spectra, (d) *g*_CD_ spectra of (*S*‐MBA)_2_PbI_4_ films without and with an AlF_3_ interlayer measured under sample flipping. Front and Back represent the apparent CD and the corresponding *g*_CD_, while Semi‐Sum represents the CD*_genuine_* and its corresponding *g*_CD_. Lines and shaded areas represent the mean ± SD for at least three independently prepared films (*n* ≥ 3).

The first term represents the CD*_genuine_* signal originating from the intrinsic chiroptical properties of the material. The second term accounts for the LDLB contribution arising from the interference between linear dichroism and linear birefringence. The third term is associated with the interaction between LD and elliptically polarized light caused by imperfections in the polarization modulation of the instrument. As shown in Figure S12, S13, the apparent CD and corresponding *g*_CD_ values showed no pronounced angular dependence upon rotating the samples in 60° increments. Therefore, whereas the CD*_genuine_* and LDLB terms correspond to reproducible sample‐dependent optical responses, the third term is treated as an instrumental artifact. CD*_genuine_* can be obtained by averaging the CD measured from the front and back sides of the film, by using the following equation:

(2) $$\;CD_{genuine}=\frac{1}{2}\;\left(CD_{front}+CD_{back}\right)$$

The LDLB contribution has an opposite sign, when the CD is measured from the front and back sides after flipping the film. The LDLB contributions can be estimated by the following equation:

(3) $$LDLB=\frac{1}{2}\;\left(CD_{front}-CD_{back}\right)$$

To separate the CD*_genuine_* and LDLB contributions, CD spectra were measured from both the front and back sides of each film after flipping the sample.

The front side (*R*/*S*‐MBA)_2_PbI_4_ films exhibited vertically mirrored negative and positive CD signal near the excitonic absorption region (530 nm).

CD signals of both front‐side and back‐side (*R*‐MBA)_2_PbI_4_ films with AlF_3_ layers as high as ∼130 mdeg were detected, while the CD signals of the only (*R*‐MBA)_2_PbI_4_ films were small, ∼30 mdeg at ∼520 nm (Figure 3a). The *g*_CD_ was defined by normalizing the CD signal with the corresponding optical absorbance, as shown in the following equation:

(4) $$\;g_{CD}=\frac{CD\left(\mathrm{mdeg}\right)}{32980\;\times Absorbance}$$

Accordingly, the (*R*‐MBA)_2_PbI_4_ films with a 30‐nm‐thick AlF_3_ layer exhibited the highest *g*_CD_ of ∼0.0016 (0.16%), representing an approximately 3.8‐fold increase compared with ∼0.00043 (0.04%) for the films without the AlF_3_ layer, while the films with 10‐ and 50‐nm‐thick AlF_3_ layers showed *g*_CD_ values of ∼0.0011 (0.11%) (Figure 3b and Figure S12). A similar trend was observed for the (*S*‐MBA)_2_PbI_4_ films (Figure 3c and 3d). These results implied that the introduction of 30‐nm‐thick AlF_3_ layers effectively increased the chiroptical properties of (*R*/*S*‐MBA)_2_PbI_4_ chiral perovskite films. However, the CD*_genuine_* signals exhibited little variation upon the introduction of the AlF_3_ layer, whereas the apparent CD signals were significantly enhanced. As shown in Figure S14, the AlF_3_‐incorporated films exhibited a more pronounced LDLB contribution than the pristine films, suggesting that the observed increase in the measured CD response is primarily associated with the increased LDLB contribution rather than with an enhancement of the intrinsic chiroptical properties [27, 55, 56]. For a more detailed examination, the CD, *g*_CD_, and LDLB spectra obtained from three independently prepared (*R/S*‐MBA)_2_PbI_4_ films without and with the AlF_3_ interlayer are separately presented in Figure S15. These measurements showed consistent CD, *g*_CD_, and LDLB characteristics with those presented in Figure 3 and Figure S14. To investigate the origin of this increase in CD response in more detail, Williamson–Hall analysis was performed on the XRD peaks (Figure S16). It revealed that the microstrain of the AlF_3_‐incorporated (*R*‐MBA)_2_PbI_4_ films increased to 0.938 × 10^−3^ from 0.375 × 10^−3^ for the pristine films, corresponding to an approximately 2.5‐fold increase. These results suggest that the enhanced measured CD response may be associated with the change in microstrain induced by the introduction of the AlF_3_ interlayer [22, 57]. The CPL‐PL properties were further investigated by measuring the left‐ and right‐handed circularly polarized emission components from the (*R*/*S*‐MBA)_2_PbI_4_ films (Figure S17). The (*S*‐MBA)_2_PbI_4_ and (*R*‐MBA)_2_PbI_4_ films exhibit CPL signals with opposite signs, showing negative and positive responses, respectively.

Both (*R*/*S*‐MBA)_2_PbI_4_ films exhibited CPL emission centered at ∼550 nm. Notably, the introduction of the AlF_3_ layer led to a substantial enhancement of the CPL response, producing signals as high as ∼200 mdeg, nearly twice those observed for the corresponding control films (∼100 mdeg). Consistent with this behavior, the *g*_lum_ values increased to ∼0.03 for both AlF_3_‐integrated perovskite films, compared with 0.015 and 0.020 for the pristine (*R* ‐MBA)_2_PbI_4_ and (*S*‐MBA)_2_PbI_4_ films, respectively. Additional thickness‐dependent CPL‐PL measurements showed that the 30 nm AlF_3_ sample exhibited relatively high CPL and corresponding *g*_lum_ values, while lower responses were observed for the 10 and 50 nm samples (Figure S18). The enhanced apparent CD and CPL‐PL responses may primarily be attributed to AlF_3_‐interlayer‐induced changes in lattice microstrain, rather than an enhancement of the intrinsic chiral selectivity of the perovskite films.

### 2.4 Characterization of Perovskite Film Stability With AlF_3_ Layer

To investigate the effect of the AlF_3_ layer on the stability of the perovskite films, the degradation behavior of the perovskite films was monitored under ambient conditions (23% relative humidity: RH, 25°C). Figure 4a,b present the bottom‐side view PL‐optical microscopy (PL‐OM) images of (*R*‐MBA)_2_PbI_4_ films without and with the AlF_3_ layer, respectively. The ambient‐condition stability test PL‐OM results for the (*S*‐MBA)_2_PbI_4_ films are also presented in Figure S19. The perovskite films were deposited on 30‐nm‐thick AlF_3_‐coated or pristine glass substrates and flipped over for PL‐OM measurements (Figure S20). After 4 h of ambient exposure, numerous dot‐like degradation regions appeared in the control perovskite film without the AlF_3_ layer. The degradation became substantially more pronounced after 14 h, whereas only a few degradation regions were observed in the perovskite film with AlF_3_ film. These results demonstrate that the AlF_3_ layer acts as an effective protective layer, suppressing the degradation of perovskite films, leading to enhanced environmental stability of the perovskite films [43]. Additional Water contact angle (WCA) measurements showed that the AlF_3_‐deposited substrates exhibited higher WCA values than the bare substrate (Figure S21), supporting the possible protective role of the AlF_3_ layer against moisture. These observations indicate that the introduction of the AlF_3_ layer significantly improves the environmental stability of the perovskite films.

**FIGURE 4:**
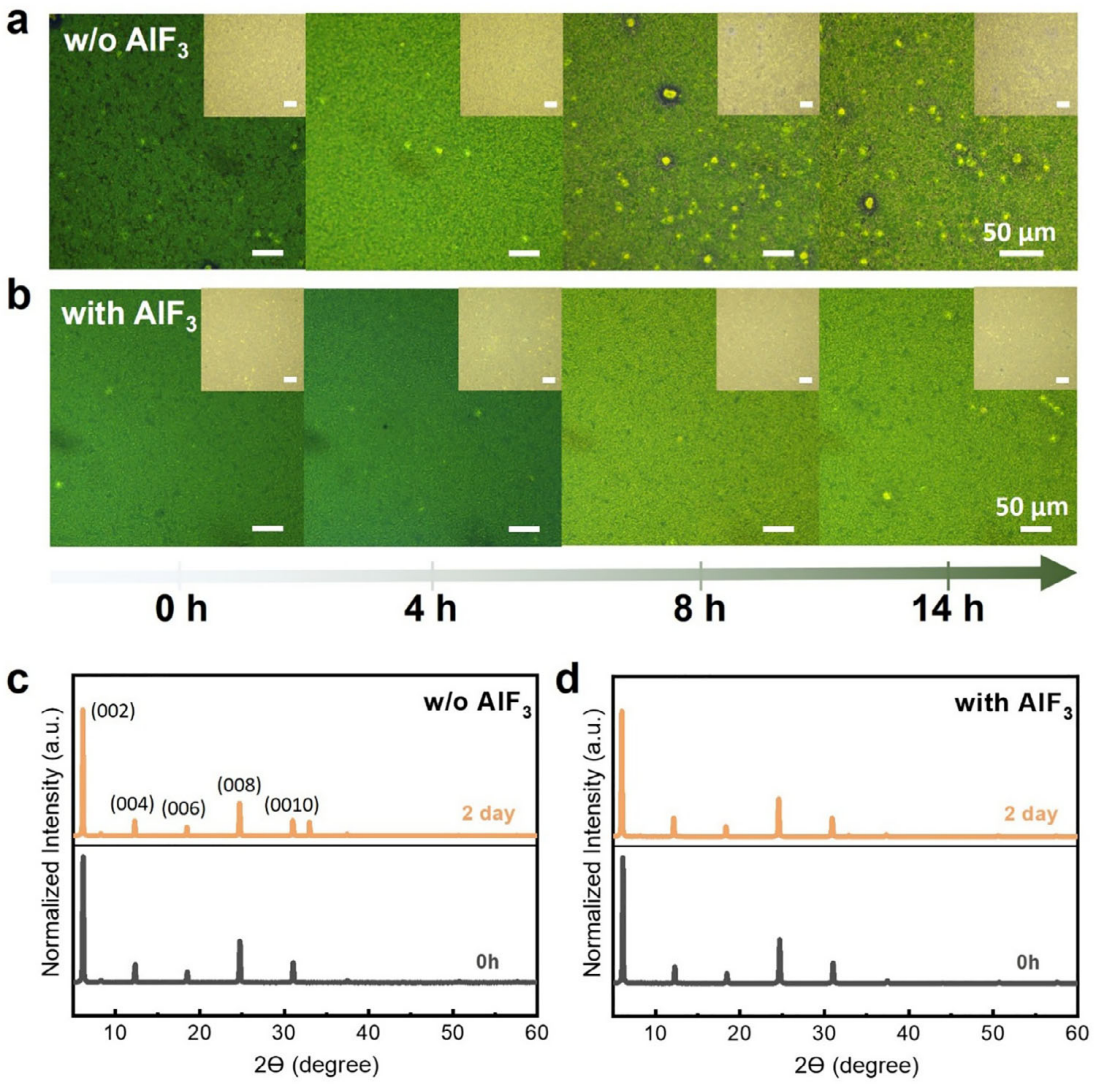
(a,b) PL‐OM Images for stability test for (*R*‐MBA)_2_PbI_4_ (Inset: OM Image) (a) without and (b) with an AlF_3_ interlayer under ambient condition. (c,d) XRD spectra for stability test for (*R*‐MBA)_2_PbI_4_ (Inset: OM Image) (c) without and (d) with an AlF_3_ interlayer under ambient condition.

Figure 4c,d, and Figure S22 shows the XRD patterns of the (*S*‐MBA)_2_PbI_4_ and (*R*‐MBA)_2_PbI_4_ films stored under ambient conditions for up to 2 days (48 h). The perovskite film incorporating the AlF_3_ layer exhibited negligible changes in its XRD pattern throughout the storage period, indicating excellent structural stability. In contrast, an unidentified diffraction peak appeared at approximately 33° in the control film without AlF_3_ after ambient exposure. The emergence of this additional peak indicates structural changes associated with perovskite degradation, further supporting the role of the AlF_3_ layer in improving film stability under ambient conditions.

### 2.5 Characterization of CPL Perovskite PDs With AlF_3_ Layer

To investigate the potential of chiral perovskite film with AlF_3_ layer in CPL optoelectronic applications, we fabricated CPL PDs by depositing gold electrode on the perovskite film (Figure 5a). The CPL PDs had a lateral device structure and device measurements were performed at room temperature in vacuum condition. In this device configuration, photogenerated carriers are transported laterally within the perovskite layer and collected directly by the top electrodes without traversing the insulating AlF_3_ interlayer. Therefore, the AlF_3_ interlayer does not directly hinder carrier extraction but primarily serves as an interfacial growth‐modulating and passivating layer. After the 505 nm laser emission passed through a linear polarizer and a quarter‐wave plate, continuous CPL was generated and irradiated onto the device. The *I–V* characteristics of (*R*/*S*‐MBA)_2_PbI_4_ CPL PDs without and with AlF_3_ layer under dark conditions, right‐handed (*R*)‐CPL and left‐handed (*L*)‐CPL illumination are shown in Figure 5b‐e. Both (*R*‐MBA)_2_PbI_4_ CPL PDs, without and with the AlF_3_ layer, exhibited higher photocurrents under *R*‐CPL illumination than under *L*‐CPL illumination, with a larger *R*‐CPL/*L*‐CPL photocurrent disparity in the PDs with AlF_3_ layer demonstrating significantly enhanced CPL distinguishability (Figure 5b,c). Similarly, the (*S*‐MBA)_2_PbI_4_ CPL PDs exhibited higher photocurrents under *L*‐CPL illumination than under *R*‐CPL illumination, and the difference was further amplified by the introduction of the AlF_3_ layer (Figure 5d,e).

**FIGURE 5:**
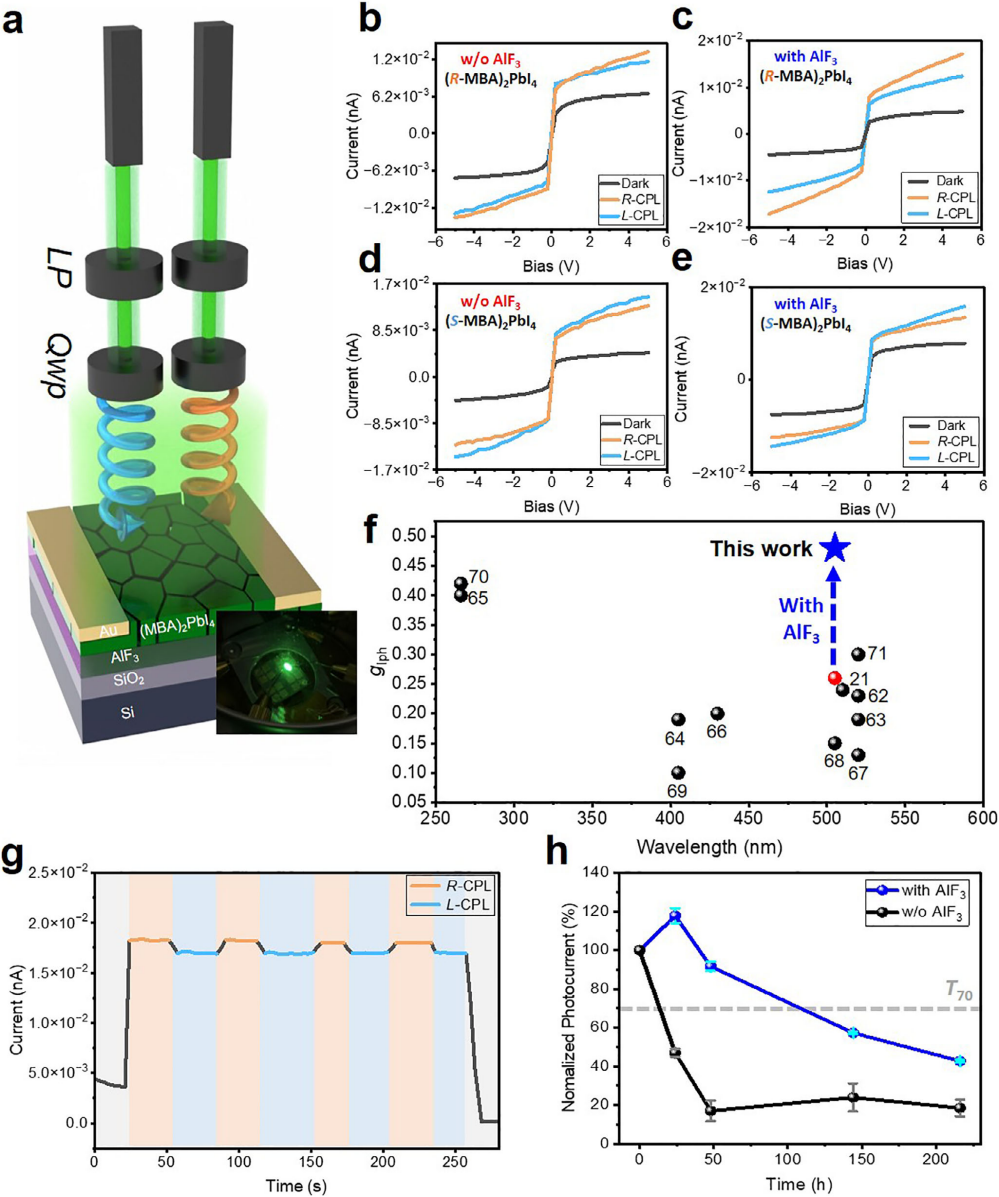
(a) Schematic illustration of the (*R/S*‐MBA)_2_PbI_4_ CPL‐PDs incorporating an AlF_3_ interlayer (inset: photograph of the photocurrent measurement setup). (b, c) *I–V* characteristics of (*R*‐MBA)_2_PbI_4_ CPL‐PDs (b) without and (c) with an AlF_3_ interlayer measured under dark conditions and green *R*‐CPL and *L*‐CPL illumination (Intensity: 235 µW cm^−2^, λ = 505 nm). (d, e) *I–V* characteristics of (*S*‐MBA)_2_PbI_4_ CPL‐PDs without and with an AlF_3_ interlayer, respectively, under dark conditions and green *R*‐CPL and *L*‐CPL illumination (Intensity: 235 µW cm^−2^, λ = 505 nm). (f) Comparison of the *g_Iph_* factors of (*R*‐MBA)_2_PbI_4_ CPL‐PDs with and without an AlF_3_ interlayer and previously reported lateral‐type 2D perovskite CPL PDs. (g) Photo‐switching characteristics of AlF_3_‐incorporated CPL‐PDs measured at a bias voltage of 5 V under alternating *R*‐CPL and *L*‐CPL illumination. (h) Photocurrent stability of CPL‐PDs without and with an AlF_3_ interlayer, evaluated at 25°C, 23% RH. (Error bars represent the standard deviation obtained from at least five independent devices.).

In order to quantify the distinguishability of CPL detection, we defined the anisotropy factor for photocurrent (*g*_Iph_) as:

(5) $$g_{Iph}=2\frac{I_{\mathrm{ph}}^{R}-I_{\mathrm{ph}}^{L}}{I_{\mathrm{ph}}^{R}+I_{\mathrm{ph}}^{L}}$$

where $I_{\mathrm{ph}}^{R}$ and $I_{\mathrm{ph}}^{L}$ represent the photocurrent under *R*‐CPL and *L*‐CPL illumination. The *g*_Iph_ of (*R*‐MBA)_2_PbI_4_ and (*S*‐MBA)_2_PbI_4_ CPL PDs with AlF_3_ layers is calculated to be 0.48 and 0.34 at 5 V bias voltage, which is larger than that of the (*R*‐MBA)_2_PbI_4_ CPL PDs without AlF_3_ (*g*_Iph_ = 0.26 and 0.18). The increased *g*_Iph_ of PDs with the AlF_3_ interlayer might be attributed to the enhanced differential absorption between *R*‐ and *L*‐CPL resulting from the increased apparent CD, together with the long spin lifetimes originating from their excellent crystalline nature [21, 58, 59, 60, 61].

Notably, the AlF_3_‐incorporated (*R*‐MBA)_2_PbI_4_ CPL PD developed in this work exhibited the highest *g_I_*_ph_ value among previously reported chiral 2D perovskite‐based lateral CPL PDs (Figure 5f) [21, 62, 63, 64, 65, 66, 67, 68, 69, 70, 71]. The photo‐switching characteristic of (*R*‐MBA)_2_PbI_4_ CPL PDs under repeated *R*‐ and *L*‐CPL illumination also show stable current output signals (Figure 5g).

Moreover, to investigate the impact of the AlF_3_ underlayer‐induced improvement on device stability, long‐term photocurrent stability measurements were performed for CPL PDs without and with the AlF_3_ layer under ambient conditions (23% RH, 25°C). As shown in Figure 5h, the pristine CPL PDs exhibited a rapid decrease in photocurrent from the initial stage of exposure, whereas the AlF_3_‐incorporated CPL PDs showed a much slower degradation behavior along with enhanced operational stability. In particular, the *T*_70_ value (defined as the time required for a 30% decrease in the initial photocurrent) increased from 12 h for the pristine devices to 112 h for the AlF_3_‐based CPL PDs, representing a 9.3‐fold improvement in operational lifetime. This result implied that the AlF_3_ interlayer effectively enhances the environmental and long‐term operational stability of chiral perovskite CPL PDs. To further evaluate the practical reliability of the CPL PDs under harsher conditions, additional long‐term photocurrent stability measurements were performed at 60% RH and 60°C (Figure S23). Under both elevated‐humidity and thermal‐aging conditions, the AlF_3_‐incorporated CPL PDs consistently exhibited longer *T*_70_ lifetimes than the pristine devices. Specifically, the *T*_70_ values increased from 19 to 23 h under 60% RH and from 17 to 27 h under 60°C. These results further demonstrate that the AlF_3_ interlayer effectively enhances the environmental and thermal stability of chiral perovskite CPL PDs by improving the film morphology and suppressing grain boundary and defect mediated degradation pathways.

We also investigated the photodetection characteristics under linearly polarized light, where the AlF_3_‐based PDs exhibited a *R* of 54 mA W^−1^, a *D* ^*^ of 1.68 × 10^11^ Jones, and stable photo‐switching behavior (Supplementary Note 1 and Figure S24).

## 3 Conclusion

In summary, we demonstrated a simple yet effective inorganic interface‐engineering strategy for enhancing the apparent chiroptical properties, device performance, and operational stability of chiral perovskite CPL PDs through the introduction of an ALD‐grown AlF_3_ underlayer. The highly dense AlF_3_ film provided a fluorine‐rich interface that promoted grain growth and defect passivation, resulting in enlarged grain sizes, reduced defect densities, and improved crystalline ordering in the chiral (*R/S*‐MBA)_2_PbI_4_ films. Consequently, the AlF_3_‐integrated perovskite films exhibited significantly enhanced chiroptical properties, with the CD and CPL‐PL *g*‐factors increased by approximately 3.8‐fold and 2‐fold increases, respectively, 2‐folds compared with those of pristine films. The improved film quality further translated into superior CPL PD performance, yielding a photocurrent g‐factor of 0.48, corresponding to an 84% enhancement in CPL discrimination capability compared to that without AlF_3_ underlayer. In addition, the AlF_3_ underlayer effectively suppressed ambient‐induced degradation, leading to a remarkable 9.3‐fold increase in the *T*_70_ operational lifetime of the PDs. These results establish ALD‐grown AlF_3_ as a promising inorganic passivation platform for simultaneously addressing the chiroptical, optoelectronic, and stability limitations of chiral perovskite devices, providing a viable pathway toward practical CPL optoelectronic technologies.

## 4 Experimental Section

### 4.1 Materials

*R*‐MBA(99% optical purity >97%), *S*‐MBA(98% optical purity >97%), and lead(II) acetate trihydrate ((CH_3_COO)_2_Pb·3H_2_O, 99.5%) were purchased from GreatCell Solar. 55–57 wt% aqueous hydroiodic acid (HI) solution, anhydrous N,N‐dimethylformamide (DMF), and anhydrous dimethyl sulfoxide (DMSO) were purchased from Sigma‐Aldrich. Trimethylaluminum (TMA, electronic grade) and hydrogen fluoride–pyridine complex (HF–pyridine, ∼70 wt% HF) were used as ALD precursors. Anhydrous toluene was used as a washing solvent during crystal purification. All reagents were of analytical grade and used without further purification.

### 4.2 Synthesis of Chiral Hybrid Perovskite (R/S‐MBA)_2_PbI_4_

(*R/S*‐MBA)_2_PbI_4_ crystals were synthesized by dissolving lead(II) acetate trihydrate (0.759 g) and *R*‐MBA or *S*‐MBA (0.485 g) in 10 mL of aqueous HI solution in a glass vial. The precursor solution was magnetically stirred at 120°C and 400 rpm for 8 h (*R*‐MBA) or 3 h (*S*‐MBA) until a clear, homogeneous solution was obtained. The solution was then allowed to cool slowly to room temperature and left undisturbed under ambient conditions for 48 h to induce crystallization, yielding orange needle‐shaped crystals. The resulting crystals were collected by centrifugation at 15000 rpm for 20 min and washed repeatedly with anhydrous toluene to remove residual precursors and impurities. The purified crystals were dried in a vacuum oven at 60°C for 48 h prior to use.

### 4.3 ALD of AlF_3_ Interfacial Buffer Layers

AlF_3_ thin films were deposited by thermal ALD using a flow‐type, hot‐wall ALD system (mini‐ALD, NexusBe). Prior to deposition, all substrates were sequentially cleaned by ultrasonication in acetone, isopropanol, and deionized water for 20 min each, blown dry with high‐purity nitrogen gas, and immediately transferred to the ALD chamber to minimize surface contamination. TMA and HF–pyridine were used as the aluminum precursor and fluorine reactant, respectively, with both precursor canisters maintained at room temperature (∼25°C). Ultra‐high‐purity argon (Ar, 99.99%) served as both the carrier and purge gas, and the chamber pressure was maintained at approximately 1 Torr throughout the deposition.

All depositions were conducted at a substrate temperature of 125°C, which lies within the confirmed ALD process window (125°C–175°C) where self‐limiting, temperature‐independent growth behavior was verified. One ALD cycle consisted of a TMA pulse (1 s), an Ar purge (25 s), an HF–pyridine pulse (0.7 s), and a second Ar purge (25 s), yielding a growth per cycle (GPC) of approximately 0.20 nm/cycle. AlF_3_ films with target thicknesses of 10, 30, and 50 nm were deposited by controlling the number of ALD cycles, as confirmed by spectroscopic ellipsometry.

### 4.4 Structural and Morphological Analysis

The crystal structure and phase purity of the (*R/S*‐MBA)_2_PbI_4_ films were examined by XRD (Rigaku D/Max‐2200/PC) using Cu Kα radiation (λ = 1.5406 Å) operated at 40 kV and 50 mA over a 2θ range of 5°–50°. X‐ray photoelectron spectroscopy (XPS, K‐Alpha, Thermo Fisher Scientific) was performed to investigate the chemical states of the perovskite films with and without the AlF_3_ underlayer. For XPS measurements, chiral perovskite films were prepared from a 5 wt% precursor solution.

The surface morphology, grain size distribution, and cross‐sectional structure of both AlF_3_ and perovskite films were characterized by field‐emission SEM (FE‐SEM, Carl Zeiss Gemini 560). The thickness and optical constants (refractive index n and extinction coefficient k) of the deposited AlF_3_ films were determined by spectroscopic ellipsometry (UVISEL+, Horiba) over a photon energy range of 0.5–6.5 eV with a spectral step of 0.05 eV. The surface potential distribution of the CHP films was mapped by Kelvin probe force microscopy (KPFM, Bruker Dimension Icon) operated in tapping mode.

### 4.5 Optical and Chiroptical Characterization

Optical absorption spectra were recorded by UV–Vis spectrophotometry (PerkinElmer Lambda 365) over 450–700 nm, and chiroptical properties were characterized by CD spectroscopy (Jasco J‐1700) over 300–700 nm. Steady‐state PL spectra were collected using a fluorescence spectrometer (Horiba Fluorolog‐QM) with an excitation wavelength of 395 nm. Spatial PL uniformity and humidity‐induced degradation behavior were evaluated by conventional PL‐OM under 23% RH. CPL‐PL spectra were recorded using a CPL spectrometer under excitation at 390 nm to evaluate the chiroptical emission characteristics of the films. Time‐resolved photoluminescence (TR‐PL) measurements were performed using a time‐correlated single‐photon counting (TCSPC) system under pulsed excitation at 375 nm to investigate carrier recombination dynamics.

### 4.6 Fabrication of CPL Perovskite PDs

AlF_3_‐coated Si substrates were prepared using the ALD process described above. Prior to perovskite film deposition, the AlF_3_‐deposited substrates were treated with UV/O_3_ for 10 min to improve surface wettability. The synthesized (*R/S*‐MBA)_2_PbI_4_ crystals were dissolved in a mixed solvent of anhydrous DMF and DMSO (volume ratio 10:1) at a concentration of 20 wt%. The precursor solution was spin‐coated onto the substrates at 2000 rpm for 30 s, followed by thermal annealing at 100°C for 10 min under nitrogen atmosphere air. Identical deposition parameters were applied for both *R*‐ and *S*‐type precursor solutions to enable direct comparison of chiroptical properties. Finally, Cr/Au (5/100 nm) electrodes were deposited by thermal evaporation through a shadow mask, defining an interdigitated electrode channel with a width of 500 µm and a gap length of 50 µm between adjacent electrodes.

### 4.7 Device Characterization

The current–voltage (*I*–*V*) characteristics and real‐time photo‐switching behavior of the CPL perovskite PDs were measured in a vacuum chamber using a Keithley 4200‐SCS semiconductor parameter analyzer equipped with a 505 nm green laser source. A linear polarizer(Thorlabs) and a quarter wave plate (Thorlabs) were used for CPL light generation. For stability, the CPL PDs were stored in a closed chamber under ambient conditions (23% RH, 25°C).

## Conflicts of Interest

The authors declare no conflicts of interest.

## Supporting information




**Supporting File**: smtd71019‐sup‐0001‐SuppMat.docx.

## Data Availability

The data that support the findings of this study are available from the corresponding author upon reasonable request.
