# Supplementary material for: Enhancing Chiroptical Selectivity and Device Stability of Chiral Perovskite Photodetectors Via Interfacial AlF3 Passivation
Source: Small Methods. 2026 Sep 7;10(19):e71019. doi: 10.1002/smtd.71019 (PMC13646045; doi:10.1002/smtd.71019)
Supplement: Supplementary file 1 [file SMTD-10-e71019-s001.docx]

Supporting Information

Enhancing Chiroptical Selectivity and Device Stability of Chiral Perovskite Photodetectors via Interfacial AlF_3_ Passivation

Hyejin Lim^1^^†^, Su Hye Jeong^2†^, Chae Bin Lee^2^, Yousang Won^3^, Seungwon Lee^4^, Inho Song^4^, Wooseok Song^1^, Ki‐Seok An^1^, Sungwoong Park^1^, Seungju Lee^2^, Soonmin Yim^1*^, Yoon Ho Lee^2*^, Saewon Kang^1*^

^1^ Thin Film Materials Research Center, Korea Research Institute of Chemical Technology (KRICT), 141 Gajeong-ro, Yuseong-gu, Daejeon, 34114, Republic of Korea

^2^Department of Materials Science and Engineering, Sungshin Women’s University; Seoul 01133, Republic of Korea

^3^Samsung Electronics Co., Ltd., Suwon 16677, Republic of Korea

^4^Department of Chemical Engineering, Chung-Ang University (CAU), Seoul 06974, Republic of Korea

^†^ These authors contributed equally: Hyejin Lim, Su Hye Jeong.

Corresponding author: Saewon Kang; skang@krict.re.kr, Yoon Ho Lee; yoonhlee@sungshin.ac.kr, Soonmin Yim; s.yim@krict.re.kr

**Keywords:** atomic layer deposition, chiral perovskite layer, perovskite photodetector, stability

**Fig S1.** (a) Top and (b) cross-section view SEM images of AlF_3_ films.

**Fig S2.** Low-magnification SEM images of (*R*-MBA)_2_PbI_4_ (a) without and (b) with an AlF_3_ interlayer.

**Fig S3.** SEM images (left) and grain size distributions (right) of (*R*-MBA)_2_PbI_4_ without an AlF_3_ interlayer and with AlF_3_ interlayers of various thicknesses (10, 30, and 50 nm) in the dense film region.

**Fig S4**. XPS spectra of the (*R*-MBA)_2_PbI_4_ films without and with AlF_3_ interlayer: (a) survey spectra, (b) F 1s, (c) Pb 4f, and (d) I 3d core-level spectra.

**Fig S5.** (a) UV–vis absorption and (b) PL spectra of (*S*-MBA)_2_PbI_4_ films without and with an AlF_3_ interlayer.

**Fig S6.** (a) UV–vis absorbance spectra of the without and with an AlF_3_ interlayer.

**Fig S7.** (a) TR-PL decay curves of (*S*-MBA)_2_PbI_4_ films without and with an AlF_3_ interlayer.

**Fig S8.** TR-PL decay curves of (*R*-MBA)_2_PbI_4_ films with different AlF_3_ interlayer thicknesses (0, 10, 30, and 50 nm).

**Fig S9.** GIWAXS patterns of (*R*-MBA)_2_PbI_4_ films without (left) and with (middle) an AlF_3_ interlayer, and the corresponding out-of-plane GIWAXS line-cut profiles extracted from the 2D GIWAXS patterns (right), measured at incident angles of (a) 0.2°, (b) 0.4°, (c) 0.5°, (d) 0.7°, (e) 0.9°, and (f) 1.1°.

**Fig S10.** (a) KPFM topography images of (*R*-MBA)_2_PbI_4_ films without (left) and with (right) an AlF_3_ interlayer.

**Fig S11.** KPFM topography images (a) without and (b) with an AlF_3_ interlayer. KPFM surface potential maps of (*S*-MBA)_2_PbI_4_ films (c) without and (d) with an AlF_3_ interlayer. (e) Surface potential line profiles extracted from the corresponding KPFM measurements.

**Fig S12**. Front-side (a) CD (b) *g*_CD_ values, and back-side (c) CD and (d) *g*_CD_ values of (*R*-MBA)_2_PbI_4_ films with different AlF_3_ interlayer thicknesses (0, 10, 30, and 50 nm), measured at different sample rotation angles.

**Fig S13**. CD (left) and *g*_CD_ (right) spectra of (*R*-MBA)_2_PbI_4_ films without and with an AlF_3_ interlayer measured at different sample rotation angles: (a) 0°, (b) 60°, (c) 120°, (d) 180°, (e) 240°, and (f) 300°.

**Fig S14**. (a) CD*_genuine_* and (b) LDLB spectra of (*R*-MBA)_2_PbI_4_ films, and (c) CD*_genuine_* and (d) LDLB spectra of (*S*-MBA)_2_PbI_4_ films without and with an AlF_3_ interlayer. Lines and shaded areas represent the mean ± SD for at least three independently prepared films (n ≥ 3).

**Fig S15**. CD spectra (left), *g*_CD_ spectra (middle), and LDLB spectra (right) of (a–c) (*R*-MBA)_2_PbI_4_ and (d–f) (*S*-MBA)_2_PbI_4_ films without and with an AlF_3_ interlayer. Each row represents an independently prepared sample.

**Fig S16**. Williamson–Hall plots of (*R*-MBA)_2_PbI_4_ films (a) without and (b) with an AlF_3_ interlayer.

**Fig S17**. (a) CPL-PL and (b) *g*_lum_ spectra of (*S*-MBA)_2_PbI_4_ and (*R*-MBA)_2_PbI_4_ films without and with an AlF_3_ interlayer.

**Fig S18.** Front-side (a) CPL-PL and (b) *g*_lum_ values of (*R*-MBA)_2_PbI_4_ films with different AlF_3_ interlayer thicknesses (0, 10, 30, and 50 nm), measured at different sample rotation angles.

**Fig S19**. PL-OM Images for stability test for (*S*-MBA)_2_PbI_4_ (Inset:OM Image) (a) without and (b) with an AlF_3_ interlayer under ambient condition.

**Fig S20**. Schematic image of perovskite film stability PL-OM measurements system

**Fig S21**. (a) Water contact angle images and (b) corresponding water contact angle values of AlF_3_ films with thicknesses of 0, 10, 30, and 50 nm.

**Fig S22**. XRD spectra of (*S*-MBA)_2_PbI_4_ (a) without and (b) with an AlF_3_ interlayer under ambient condition.

**Fig S23.** Photocurrent stability of CPL-PDs without and with an AlF_3_ interlayer, evaluated at (a) 60% RH and (b) 60 °C. (Error bars represent the standard deviation obtained from at least five independent devices.)

**Fig S24.** (a) *I–V* characteristics, (b) *R* and *D^*^*, (c) external quantum efficiency (*EQE*), and (d) photoswitching characteristics of AlF_3_-incorporated PDs under various intensities of green light illumination (λ = 505 nm).

**Supplementary Note 1**

The *I–V* characteristics of the AlF_3_-incorporated PDs under linear polarized green light illumination (λ = 505 nm) at various light intensities are shown in **Fig. S24a**. The photocurrent gradually increased with increasing incident light intensity owing to the generation of a larger number of photocarriers under higher photon flux. The devices exhibited a clear photoresponse under all illumination conditions, indicating efficient charge generation and transport in the AlF_3_-incorporated PDs. To quantitatively evaluate the photodetection performance, the *R* was calculated using the following equation:

$R=\frac{I_{\text{light}}-I_{\text{dark}}}{P_{\text{inc}}}$	(1)

where *I*_light_ is the current under light illumination, *I*_dark_ is the current under dark condition, and P_inc_ is the incident illumination power. In addition, *D** is a key performance parameter for PDs, typically indicating the smallest detectable signal, as follows :

$D^{*}= \frac{\left( AB \right)^{1/2}}{NEP} \left( \text{cm H}\text{z}^{1/2}\text{ W}^{-1} \right)$	(2)

$NEP=\frac{{\bar{{i_{n}}^{2}}}^{1/2}}{R} \left( \text{W} \right)$	(3)

Where *A* is the effective area of the detector in cm^2^, *B* is the bandwidth, *NEP* is the noise equivalent power, and ${\bar{{i_{n}}^{2}}}^{1/2}$is the measured noise current. If shot noise from the dark current is the major factor contributing to noise limiting the *D^*^*, then *D^*^* can be simplified as [S1-3]

$D^{*}= \frac{R}{{(2\text{e}\cdot\text{I}_{\text{dark}}/A)}^{1/2}}$	(4)

Where *e* is the charge of an electron. The *R* and *D** values as a function of light intensity are presented in **Fig. S24b**.

The enhancements in *R* and *D*^*^ significantly increased as the light intensity decreased, which is a commonly observed phenomenon [S4]. The CPL PDs fabricated on the AlF_3_ underlayer achieved a *R* of 54 mA W^-1^, a *D*^*^ of 1.68 × 10^11^ Jones (under a light intensity of 38.2 μW cm^-2^)

The external quantum efficiency (*EQE*) was further calculated according to the following equation:

$EQE= \frac{{(I}_{\text{light}}-I_{\text{dark}})hc}{eP_{\text{int}}A\lambda}$	(5)

where $h$ is Planck’s constant, $c$ is the speed of light, $P_{\text{int}}$ is the incident light intensity (i.e., the incident optical power density) in the effective area, $A$ is the active area of PDs, and *λ* is the incident wavelength. As shown in **Fig. S24c**, the *EQE* exhibited a similar trend to that of *R* and *D**, reaching a maximum value of approximately 13.3 % under 38.2 μW cm^-2^ intensity illumination and gradually decreasing with increasing light intensity.

To investigate the operational stability and reproducibility of the device, photoswitching measurements were performed under periodic light on/off illumination cycles (**Fig. S24d**). The photocurrent exhibited stable and repeatable switching behavior without noticeable degradation throughout the measurement period. Furthermore, the photocurrent increased proportionally with increasing light intensity, confirming the reliable photoresponse and excellent operational stability of the AlF_3_-incorporated PDs.

**Table S1.** Comparison of the *g*_Iph_ factors of (*R*-MBA)_2_PbI_4_ CPL-PDs with and without an AlF_3_ interlayer and previously reported lateral-type 2D perovskite circularly polarized photodetectors.

| **Perovskite** | ***g*_Iph_** | **Driven Voltage (V)** | **Detecting Wavelength (nm)** | **Peformance Parameter (*R*, *D*^*^)** | **Ref.** |
| --- | --- | --- | --- | --- | --- |
| (*R*/*S*-α-PEA)_2_PbI_4_ | 0.23 | -3 | 520 | *R* = 0.6 A/W  *D*^*^ = 3.06×10^11^ Jones | 62 |
| (*R*/*S*-MBA)_2_PbI_4_ | 0.24 | 5 | 510 | *R* = 1.44 A/W  *D*^*^ = 3.9×10^12^ Jones | 21 |
| (*R*/*S*-*β*-MPA)_4_AgBiI_8_ | 0.19 | 5 | 520 | *R* = 0.05 A/W  *D*^*^ = 3.9×10^11^ Jones | 63 |
| (*R*/*S*-*β*-MPA)EAPbBr_4_ | 0.19 | 10 | 405 | *D*^*^ = 1.1×10^11^ Jones | 64 |
| (*R*/*S*-PPA)EAPbCl_4_ | 0.4 | 0 | 266 | under 5V bias  *D*^*^ = 4.7×10^7^ Jones | 65 |
| (*R*/*S*-3AMP)PbBr_4_ | 0.2 | 10 | 430 | *R* = 0.04 A/W  *D*^*^ = 2.4×10^12^ Jones | 66 |
| (*R*/*S*-BPEA)_2_PbI_4_ | 0.13  (under520 nm)/ 0.1  (under 800 nm) | 10 | 520, 800 | under 520 nm  *R* = 2×10^-3^ A/W  *D*^*^ = 3×10^11^ Jones  under 800 nm  *R* = 3.5×10^-6^ A/W | 67 |
| (*R*/*S*-α-PEA)_2_PbI_4_ | 0.15 | 5 | 505 | *R* = 47.1 A/W  *D*^*^ = 1.24×10^13^ Jones | 68 |
| (*R*/*S*-PPA)(PA)PbBr₄ | 0.1 | 10 | 405 | *R* = 0.06 A/W  *D*^*^ = 2.62×10^12^ Jones | 69 |
| (*R*/*S*-PPA)EAPbBr_4_ | 0.42 | 0 | 266 | NA | 70 |
| (*R*/*S*-*β*-MPA)_4_AgBiI_8_ | 0.3 | 0 | 520 | under 5V bias  *R* = 22×10^-6^ A/W  *D*^*^ = 1.2×10^7^ Jones | 71 |
| (*R*/*S*-MBA)_2_PbI_4_ | 0.48 | 5 | 505 | *R* = 54×10^-6^ A/W  *D*^*^ = 1.6 × 10^11^ Jones | **This work** |

NA: not available

PEA: phenethylammonium

MPA: methylphenethylaminemonium

PPA: 1-phenylpropylamine

EA: ethylammonium

AMP: aminomethyl piperidine

BPEA: 1-(4-bromophenyl)ethylammonium)

**Reference**

# [S1] X. Gong, M. Tong, Y. Xia, et al., “High-Detectivity Polymer Photodetectors with Spectral Response from 300 nm to 1450 nm,” *Science* 325 (2009): 1665–1667.

# [S2] Y. H. Lee, et al., “Large‐Scale 2D Perovskite Nanocrystals Photodetector Array via Ultrasonic Spray Synthesis,” *Advanced Materials* 37 (2025): 2417761.

# [S3] C. Ji, K. Kim, S. Oh, “High-Detectivity Perovskite-Based Photodetector Using a Zr-Doped TiOₓ Cathode Interlayer,” *RSC Advances* 8 (2018): 8302–8309.

[S4] G. Konstantatos, I. Howard, A. Fischer, et al., “Sensitive Solution-Processed Visible-Wavelength Photodetectors,” *Nature Photonics* 1 (**2007**): 531–534.
